# Supplementary material for: A Drosophila model of diabetic neuropathy reveals a role of proteasome activity in the glia
Source: iScience. 2023 May 29;26(6):106997. doi: 10.1016/j.isci.2023.106997 (PMC10291573; doi:10.1016/j.isci.2023.106997)
Supplement: Document S1. Figures S1–S5 and Tables S1 and S2 [file mmc1.pdf]

iScience, Volume *26*

Supplemental information

A *Drosophila* model of diabetic neuropathy reveals a role of proteasome activity in the glia

Mari Suzuki, Hiroshi Kuromi, Mayumi Shindo, Nozomi Sakata, Naoko Niimi, Koji Fukui, Minoru Saitoe, and Kazunori Sango

Figure S1

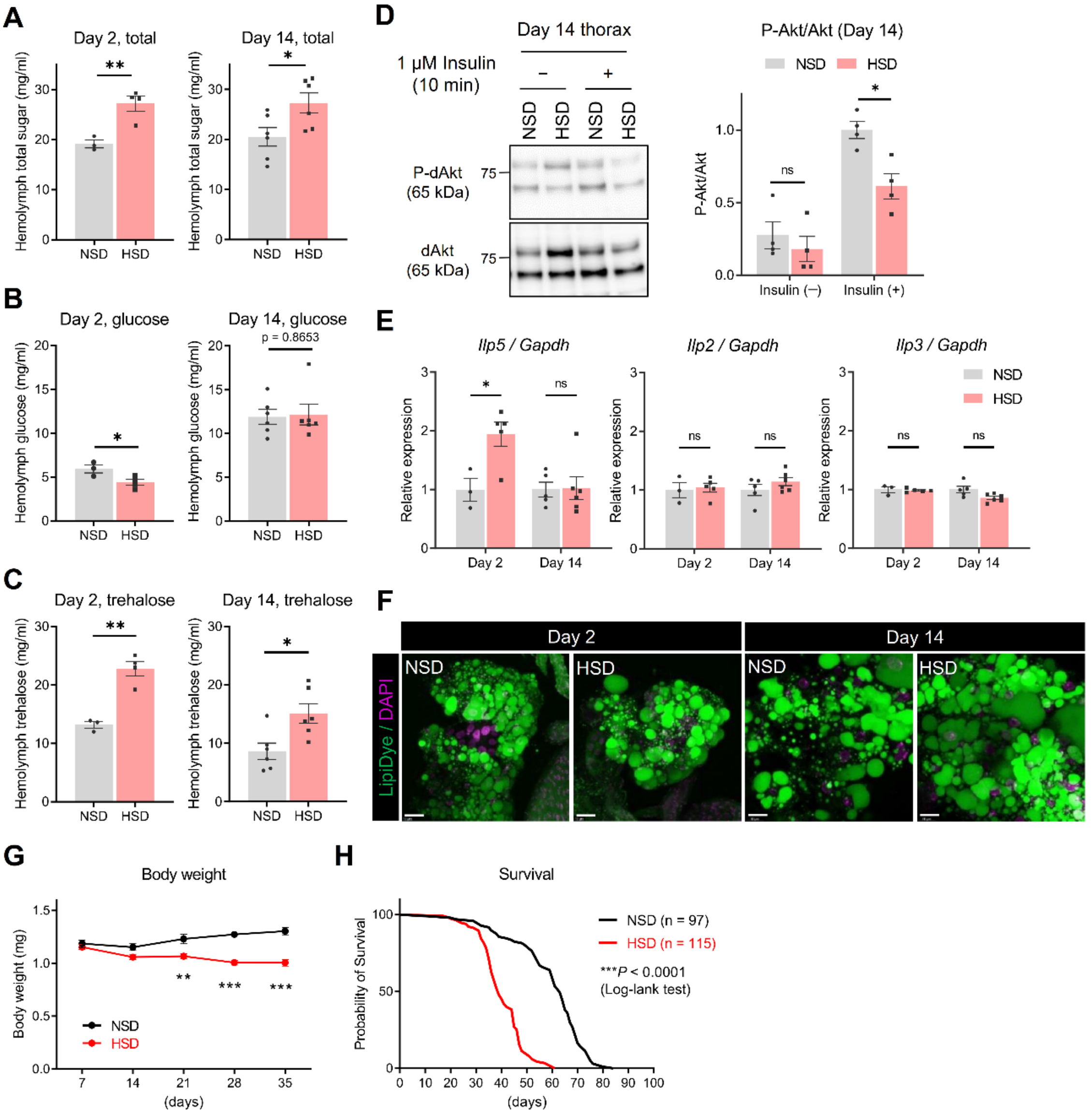

Figure S1. A high-sugar diet induced diabetes-like metabolic features in wild-type female flies, Related to Figure 1

Wild-type (WT) flies were reared on a standard cornmeal-yeast-glucose diet (normal-sugar diet, NSD), and after the eclosion, female flies were collected and divided into the NSD group and HSD (high-sugar diet, NSD with an additional 30% sucrose) group. (A–C) HSD-induced hyperglycemia. Hemolymph total sugar (glucose + trehalose) (A), glucose (B) and trehalose (C) at day 2 and 14 of HSD feeding. Unpaired *t* test (N = 3–4 for day 2, N = 6 for day 14, 19–25 flies per replicate). (D) Insulin-induced *Drosophila* Akt (dAkt) phosphorylation was reduced by HSD. Thoraxes were dissected from starved flies at 14 days and treated either with insulin or vehicle in Schneider's *Drosophila* medium. Signal intensity was quantified from western blotting for phospho-*Drosophila*

Akt (P-dAkt) and total dAkt. Two-way ANOVA post hoc Tukey's test (N = 3–4 experiments). (E) Transcript levels of *Insulin-like peptide* (*Ilp*) 5, *Ilp2*, and *Ilp3* in flies at 2 and 14 days. Two-way ANOVA post hoc Tukey's test (N = 3–6, five heads per replicate). (F) HSD causes enlargement of lipid droplets (LDs) in the abdominal fat body. LDs were stained with LipiDye. (G) HSD gradually decreased body weight. Two-way ANOVA post hoc Tukey's test (N = 5, means of 4–20 flies were calculated for each replicate). (H) HSD significantly shortened lifespan. Log-lank test ($p < 0.0001$, 97 flies for NSD, 115 flies for HSD). ns, not significant, *$p < 0.05$, **$p < 0.01$, ***$p < 0.001$.

Figure S2

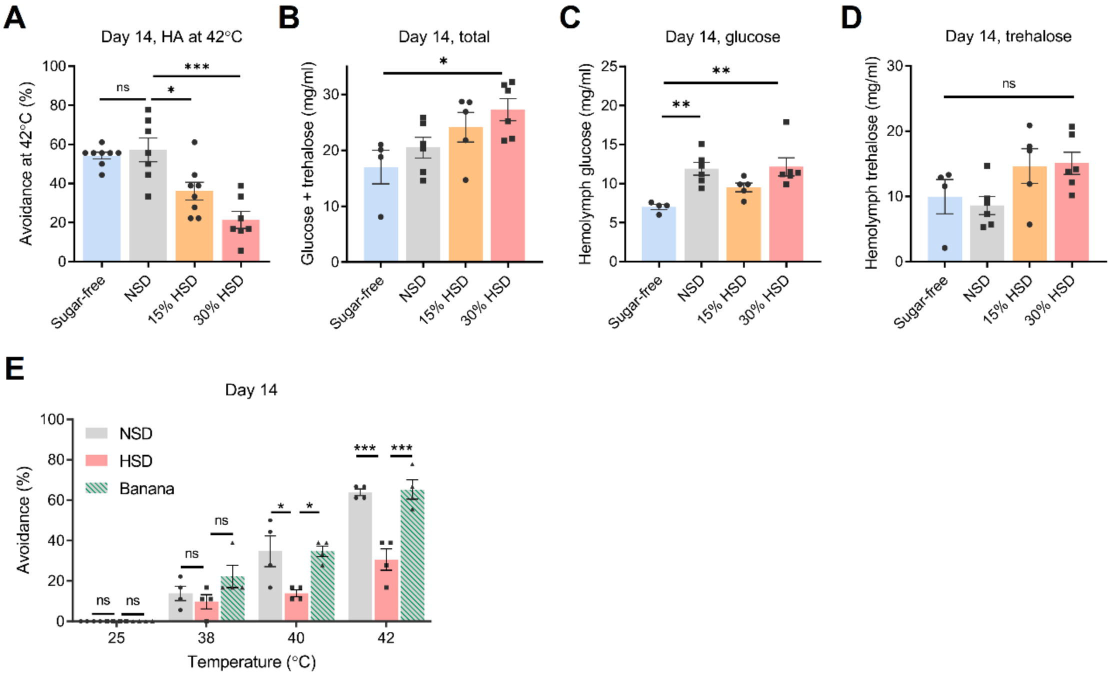

Figure S2. Dose-dependent effects of dietary carbohydrate on heat avoidance behavior and hemolymph sugar, Related to Figure 1

(A–D) Female flies were fed indicated diets for 14 days, and heat avoidance at 42°C (A) and hemolymph sugar concentrations (B–D) were analyzed. Sugar-free, NSD without glucose; 15 or 30% HSD, NSD with 15 or 30 % sucrose. One-way ANOVA post hoc Tukey's test (N = 4–8, 7–9 flies per replicate for A, 19–25 flies per replicate for B–D). (E) Flies fed banana showed similar heat avoidance profiles to flies fed NSD. Two-way ANOVA post hoc Tukey's test (N = 4, 7–9 flies per replicate). ns, not significant, *p < 0.05, **p < 0.01, ***p < 0.001.

Figure S3

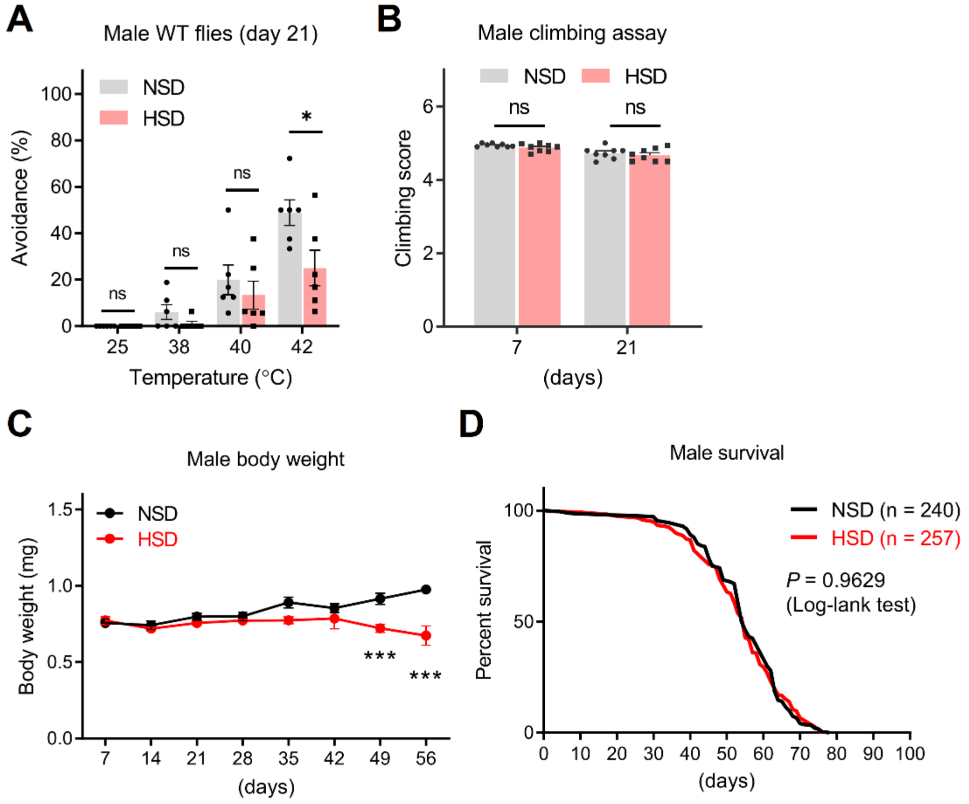

Figure S3. Effects of HSD on male WT flies, Related to Figure 1

(A) Heat avoidance test of male WT flies at day 21. Two-way ANOVA post hoc Tukey's test (N = 6, 7–9 flies per replicate). (B) Climbing assay. Two-way ANOVA post hoc Tukey's test (N = 8, 10–20 flies per replicate). (C) Body weight. Two-way ANOVA post hoc Tukey's test (N = 3–5, means of 7–20 flies were calculated for each replicate). (D) Survival curve. Log-lank test (p = 0.9629, 240 flies for NSD, 257 flies for HSD). ns, not significant, *p < 0.05, ***p < 0.001.

Figure S4

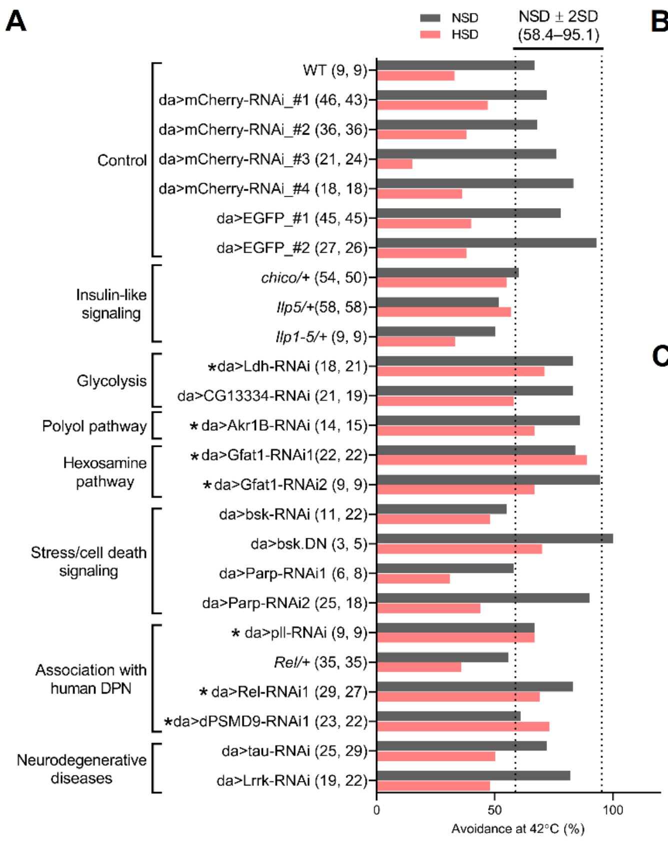

B

Fly line	% HA (NSD)	% HA (HSD)	Difference (NSD–HSD)
da>dPSMD9-RNAi1	61.0	73.0	-12.0
da>Gfat1-RNAi1	84.0	89.0	-5.0
da>pll-RNAi	67.0	67.0	0.0
da>Ldh-RNAi	83.0	71.0	12.0
da>Rel-RNAi1	83.0	69.0	14.0
da>Akr1B-RNAi	86.0	67.0	19.0
da>Gfat1-RNAi2	94.4	66.7	27.7

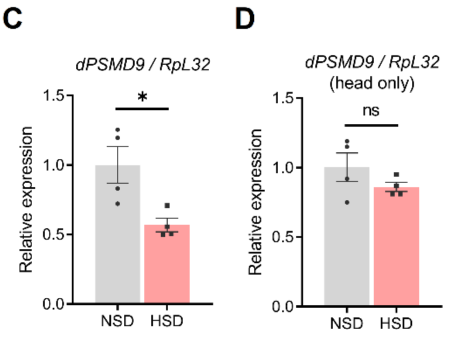

Figure S4. Candidate genetic screening for modifiers of HSD-induced heat avoidance impairment, Related to Figure 3

(A) Screening for genetic modifiers of HSD-induced heat avoidance impairment. RNAi-mediated knockdown or overexpression flies under the control of the *daughterless* (*da*)-*GAL4*, or heterozygote mutant flies were raised on NSD or HSD for 14 days after eclosion and used for the heat avoidance test. *da-GAL4>mCherry-RNAi* (da>mCherry-RNAi #1–4) and *da-GAL4>EGFP* (da>EGFP #1–2) flies were used as control. The numbers in parentheses indicate the fly numbers used for NSD or HSD, respectively. The reference range was set as 58.4%–95.1 %, and seven fly lines, both of which heat avoidance rates for NSD and HSD are included in the reference range, were identified as hits (asterisks). (B) Hit fly lines were listed in order of the decreasing means difference between NSD and HSD. (C) Expression levels of *dPSMD9* in HSD-fed flies. Total RNA was isolated from whole-bodies of 14-days-old flies. *RpL32* was used as an internal control. Student *t* test (N = 4, tow flies per replicate). (D) Expression levels of *dPSMD9* in the head of HSD-fed flies. Total RNA was isolated from heads of 14-days-old flies. *RpL32* was used as an internal control. Student *t* test (N = 4, five heads per replicate).

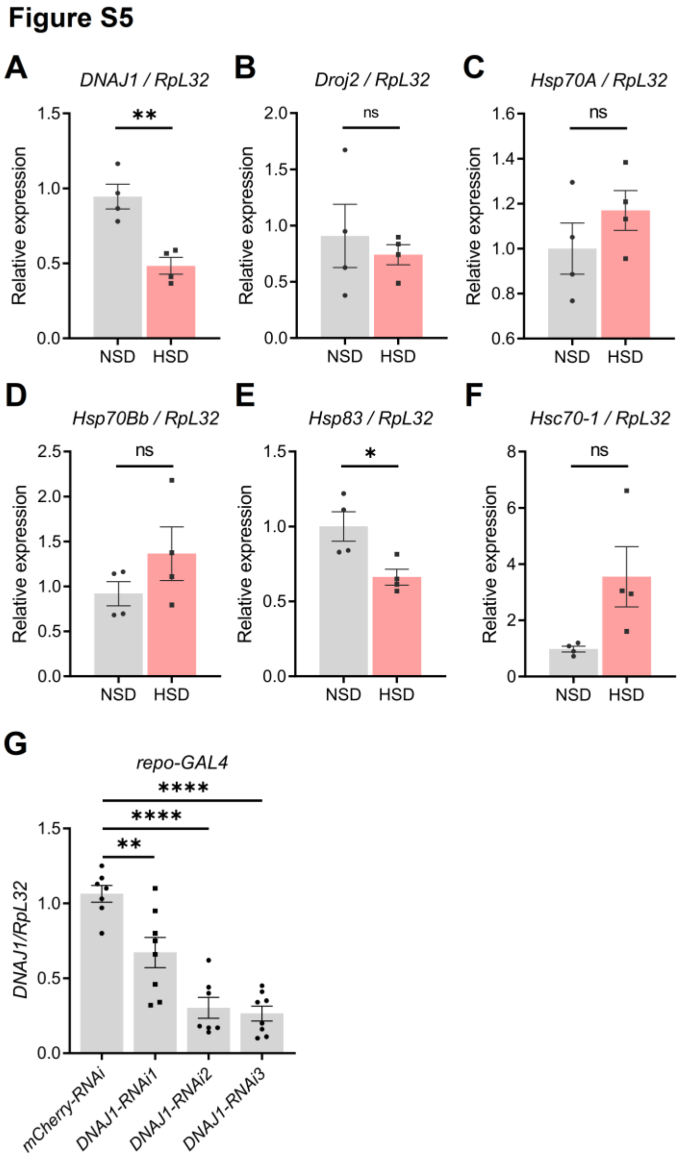

Figure S5. Expression levels of HSPs in the HSD-fed flies, Related to Figure 6

(A–F) Expression levels of HSPs in the HSD-fed flies. Total RNA was isolated from whole bodies of 14-days-old flies. *RpL32* was used as an internal control. Student *t* test for (A) *DNAJ1*, (B) *Droj2*, (C) *Hsp70A*, (D) *Hsp70Bb*, (E) *Hsp83*. Welch's *t* test for (F) *Hsc70-1*. N = 4, two flies per replicate. (G) Analysis of *DNAJ1* knockdown efficiency. RNAi-mediated knockdown was driven by the *repo-GAL4* driver. Total RNA was isolated from heads of 3- to 7-days-old flies. One-way ANOVA post hoc Tukey's test (N = 7–8, five heads per replicate).

Table S1. *Drosophila* diet recipes, Related to STAR METHODS

Cornmeal-yeast-glucose diets (per L of media)

	NSD	HSD (30%)	HSD (15%)	Sugar-free	Banana
Sucrose (Nacalai Tesque Cat# 30403-84)	–	300 g	150 g	–	
Glucose (Nacalai Tesque Cat# 16805-64)	100 g			–	
Agar (Ina Food Industry Cat# BA-10)	7.5 g				
Baker's yeast (Oriental Yeast)	35 g				
Cornmeal (Oriental Yeast)	55 g				
Bokinin* (Nacalai Tesque Cat# 06327-15)	10 mL				
Propionic acid (FUJIFILM Wako Cat# 163-04726)	5 mL				
H_2O	up to 1 L				
Total kcal	737	1933	1335	377	930
% Carbohydrate (w/v)	15.5	45.4	30.4	5.5	22.5
% Fat (w/v)	0.5				0.2
% Protein (w/v)	1.8				1.1

*10 % (w/v) butyl 4-hydroxybenzoate in ethanol.

Instant blue food (per φ24×H95mm vial)

	NSD	HSD
Formula 4-24® Instant *Drosophila* Medium, Blue food (Carolina Biological Supply Cat# 173214)	1.2 g	
H_2O	4 mL	–
30% sucrose in H_2O	–	4 mL

**For saccharides experiment (Fig. 1D), the indicated saccharide solution was used instead of 30% sucrose solution.

***Vehicles (DMSO and ethanol), pioglitazone, metformin, ixazomib, and RU486 stock solutions were added to H_2O or 30% sucrose in H_2O.

Table S2. Candidate genes for genetic screening, Related to Figure 3

Candidate genes were selected by functions in glucose metabolism (insulin-like signaling, glycolysis, polyol pathway, and hexosamine pathway), stress and cell death signaling, association with human DPN, and neurodegenerative diseases. Asterisks indicate human genes that have been reported to be associated with DPN.

Features	Fly gene	Human orthologs	Known functions, related reference(s)
Glucose metabolism	*chico*	*IRS1*	A substrate of the Insulin-like receptor; control of cell size and growth; related to HSD-induced cardiomyopathy in *Drosophila* [S1]
	Insulin-like peptide 5 (*Ilp5*)	*Insulin,* *Insulin-like growth factor*	A peptide involved in the insulin signaling pathway
	Lactate dehydrogenase (*Ldh*)	*LDHA,* *LDHB*	Involved in glycolysis, gluconeogenesis, and pyruvate metabolism
	CG13334	*LDHAL6A/B/C*	Predicted to enable L-lactate dehydrogenase activity
	Aldo-keto reductase 1B (*Akr1B*)	*AKR1B1**, *AKR1B10,* *AKR1B15,* *AKR1D1,* *AKR1C4,* *AKR1C2*	Predicted to aldose reductase, indanol dehydrogenase, and D-threo-aldose 1-dehydrogenase activity; Human ortholog *AKRB1* implicated in several diseases including DPN [S2−4]
	Glutamine-fructose-6-phosphate aminotransferase 1 (*Gfat1*)	*GFAT2,* *GFAT1*	Predicted to enable carbohydrate derivative binding activity and glutamine-fructose-6-phosphate transaminase activity; related to HSD-induced cardiomyopathy and podocyte dysfunction in *Drosophila* [S1, 5]
Stress and cell death signaling	*basket (bsk)*	*MAPK8,* *MAPK10*	Encodes a serine/threonine-protein kinase, a key component of JNK pathway; regulation of stress response
	Poly-(ADP-ribose) polymerase (Parp)	*PARP1**	Encodes a nuclear enzyme modifying acceptor proteins through assembly of poly(ADP-ribose) polymers; Human ortholog is implicated in DPN [S6]
Association with human DPN	*Pelle (pll)*	*IRAK4*	Encodes a serine-threonine protein kinase that functions in the Toll pathway. Human Toll-like receptor 4 is implicated in DPN [S7]
	Relish (Rel)	*NFKB1*	Encodes a transcription factor and the downstream component of the immune deficiency pathway, which regulates the antibacterial response. Human ortholog mediate a signal from toll-like receptor
	CG9588 (*PSMD9*)	*PSMD9**	A chaperone during the assembly of the 26S proteasome. Human ortholog is implicated in DPN [S8, 9]
Neurodegenerative diseases	*tau*	*MAPT*	Microtubule-associated protein; human ortholog protein is related to several neurodegenerative diseases such as Alzheimer's disease, Pick's disease, frontotemporal dementia, cortico-basal degeneration, and progressive supranuclear palsy
	Leucine-rich repeat kinase (*Lrrk*)	*LRRK1,* *LRRK2*	Encodes a large Ser/Thr kinase involved in mRNA translational control, human ortholog protein LRRK2 is related to Parkinson's disease

Supplemental references

S1. Na, J., Musselman, L.P., Pendse, J., Baranski, T.J., Bodmer, R., Ocorr, K., and Cagan, R. (2013). A *Drosophila* model of high sugar diet-induced cardiomyopathy. PLoS genetics 9, e1003175. 10.1371/journal.pgen.1003175.

S2. Heesom, A.E., Millward, A., and Demaine, A.G. (1998). Susceptibility to diabetic neuropathy in patients with insulin dependent diabetes mellitus is associated with a polymorphism at the 5' end of the aldose reductase gene. J Neurol Neurosurg Psychiatry 64, 213-216. 10.1136/jnnp.64.2.213.

S3. Sivenius, K., Pihlajamaki, J., Partanen, J., Niskanen, L., Laakso, M., and Uusitupa, M. (2004). Aldose reductase gene polymorphisms and peripheral nerve function in patients with type 2 diabetes. Diabetes Care *27*, 2021-2026. 10.2337/diacare.27.8.2021.

S4. Gupta, B., and Singh, S.K. (2017). Association of aldose reductase gene polymorphism (C-106T) in susceptibility of diabetic peripheral neuropathy among north Indian population. J Diabetes Complications 31, 1085-1089. 10.1016/j.jdiacomp.2017.04.011.

S5. Na, J., Sweetwyne, M.T., Park, A.S., Susztak, K., and Cagan, R.L. (2015). Diet-Induced Podocyte Dysfunction in *Drosophila* and Mammals. Cell reports 12, 636-647. 10.1016/j.celrep.2015.06.056.

S6. Nikitin, A.G., Chudakova, D.A., Strokov, I.A., Bursa, T.R., Chistiakov, D.A., and Nosikov, V.V. (2008). Leu54Phe and Val762Ala polymorphisms in the poly(ADP-ribose)polymerase-1 gene are associated with diabetic polyneuropathy in Russian type 1 diabetic patients. Diabetes Res Clin Pract 79, 446-452. 10.1016/j.diabres.2007.10.020.

S7. Rudofsky, G., Jr., Reismann, P., Witte, S., Humpert, P.M., Isermann, B., Chavakis, T., Tafel, J., Nosikov, V.V., Hamann, A., Nawroth, P., and Bierhaus, A. (2004). Asp299Gly and Thr399Ile genotypes of the TLR4 gene are associated with a reduced prevalence of diabetic neuropathy in patients with type 2 diabetes. Diabetes Care 27, 179-183.

S8. Gragnoli, C. (2011). PSMD9 is linked to type 2 diabetes neuropathy. J Diabetes Complications 25, 329-331. 10.1016/j.jdiacomp.2011.06.003.

S9. Salcini, C., Sunter, G., Ozen, F., Ozer, Y., and Arslan, B.A. (2021). Proteasome Modulator 9 Gene rs14259 Polymorphism in Patients with Diabetic Polyneuropathy. Noro Psikiyatr Ars 58, 289-291. 10.29399/npa.24801.
